# Historical trends in Hawaiian game harvest and hunter participation in Hawai‘i from 1946-2008

**DOI:** 10.1371/journal.pone.0219283

**Published:** 2019-08-16

**Authors:** Christopher A. Lepczyk, Deidre J. Duffy

**Affiliations:** 1 Department of Natural Resources and Environmental Management, University of Hawai‘i at Mānoa, Honolulu, Hawaii, United States of America; 2 School of Forestry and Wildlife Sciences, Auburn University, Auburn, Alabama, United States of America; Swansea University, UNITED KINGDOM

## Abstract

At present, 21 game species have been successfully established in Hawai‘i for the purpose of recreational and subsistence hunting. However, it is unknown how these management efforts have affected hunting and recreation trends in Hawai‘i and how the patterns may parallel national data. Consequently, managers and biologists in Hawai‘i have little reliable harvest and hunting participation information on which to base current and future management goals. This study provides the first ever analysis of public hunting data in the state of Hawai‘i, and is one of only a handful nationally to investigate long-term hunting dynamics in the United States. Our goal was to understand historical hunting trends in the state of Hawai‘i in order to provide baseline information to assist in current and future management efforts. Based upon this goal, our objectives were to investigate the influence that time, location, and species have had on both game harvest and hunter participation from 1946 to 2008 across the inhabited islands of Hawai‘i. We used 62 years of data from Pittman-Robertson reports to evaluate temporal trends in game harvest and hunter participation for all species, individual species, and taxonomic groups (mammals and birds) at both state and island levels. Since 1946, trends in game harvest and hunter participation in Hawai‘i have varied widely by island and species, suggesting that game management may be most effective when approached at the island or species level. Across the state the overall harvest has declined, with only a handful of species being harvested in greater numbers over time on several islands. However, our findings do highlight inconsistencies and potential biases in harvest collection data that are critical for science-based management. In particular, because every game species in Hawai‘i has been introduced, there is a critical need to improve harvest data collection and couple it with monitoring data in order to provide management and policy recommendations and develop better conservation planning guidelines.

## Introduction

Since the end of market hunting in the United States over a century ago, recreational and subsistence hunting for game species has been a closely managed activity. Specifically, hunting records have been monitored regularly at both state and federal government levels. Beginning in 1937, states seeking aid for wildlife related activities from the federal government have been required to submit annual hunting statistics to the U.S. Fish and Wildlife Service (USFWS) under the Pittman-Robertson Federal Aid to Wildlife Restoration (P-R) program. In more recent years, states have also been required to provide the USFWS with the number of persons graduating annually from hunter-education courses. Furthermore, hunting related data have been collected nationally by the USFWS every five years since 1955 using the Fishing, Hunting, and Wildlife-Associated Recreation (FHWAR) survey (e.g., U.S. Department of the Interior Fish and Wildlife Service and Department of Commerce Census Bureau 2006 [1]). While the annual state-reported data provide a wealth of information on specific game species and harvest rates, most historical trend analyses across the US have used the FHWAR data (e.g., [2–5]).

Evaluating hunting trends has rarely utilized state-reported data due to the types of questions being investigated, data availability and access, and the fact that most analyses are simply interested in national scale trends (see [2–4]). Though the FHWAR data do provide useful insights into national trends, they provide less insight into state level issues that are critical for wildlife management actions and policies. Moreover, FHWAR data have a number of challenges associated with utilizing them at the state level. For instance, changing methodology in the FHWAR makes it problematic where detailed results are needed, such as the case with how the minimum age of hunters has changed throughout the years. In addition, recent surveys (e.g., 1991, 1996, 2006) have collected data from hunters periodically during the study year in an effort to minimize recall bias, whereas earlier surveys were issued once annually, requiring subjects to recollect data from the past year. Finally, the data collection agency and the method of data collection have been inconsistent throughout the surveys (personal interviews, telephone interviews, and mail questionnaires have all been used).

Understanding historical hunting trends is especially important when formulating appropriate management goals in Hawai‘i, where every game animal is an introduced species (Table 1; [6]). As such, no Hawaiian game species has evolved in conjunction with the native biota and there are very few natural population controls [7]. Furthermore, Hawaii’s mild climate and ample resources allow for extended breeding seasons for many game species [8].

**Table 1:** Official game species list for the state of Hawai‘i as of 2018, adapted from the State Of Hawaii, Division of Forestry and Wildlife.

Species	Island where legally hunted^a^					
	H	K	L	MA	MO	O
Mammals						
Axis deer (*Axis axis*)			X	●	●	
Black-tail deer (*Odocoileus hemionus*)		X				
Feral sheep (*Ovis aries*)	X					
Goat (*Capra hircus*)	X			X	X	X
Mouflon Sheep (*Ovis musimon*)	X		X			
Pig (*Sus scrofa*)	X	X		X	X	X
Mouflon x feral sheep hybrids	●					
Birds						
Black francolin (*Francolinus francolinus*)	X	X		●	X	
California quail (*Callipepla californica*)	X	X	●	X	X	
Chestnut-bellied sandgrouse (*Pterocles exustus*)	X					
Chukar (*Alectoris chukar*)	X	X	X	X	●	
Erckel’s francolin (*F*. *erckelii*)	X	X	●			X
Gambel’s quail (*Callipepla gambelii*)	X		X			
Gray francolin (*F*. *pondicerianus*)	X	●	X	●	X	
Green pheasant (*Phasianus versicolor*)	●	●		●		
Japanese quail (*Coturnix japonica*)	X	X	●	●	●	●
Kalij pheasant (*Lophura leucomelanos*)	X					
Mourning dove (*Zenaidura macroura*)	●					
Ring-necked pheasant (*P*. *colchicus*)	X	X	X	X	●	X
Spotted dove (*Streptopelia chinensis*)	X	X	X	X	X	X
Wild turkey (*Melagris gallopavo*)	X		X	●	●	
Zebra dove (*Geopelia striata*)	X	X	X	●	●	X

^a^H = Hawai‘i island, K = Kaua‘i, L = Lāna‘i, MA = Maui, MO = Moloka‘i, O = O‘ahu, ● = not included in this analysis due to lack of sufficient data

Historically, game populations have been controlled by both hunting and large-scale eradication efforts. However, record keeping on the release and status of Hawaiian game has been extremely fragmented and scattered, with little effort made to determine the abundance of game species in Hawai‘i [9–10]. Thus, it is unknown how hunting alone has impacted game populations in the state or whether hunting trends in Hawai‘i follow a similar pattern to national data. Consequently, managers and biologists in Hawai‘i have little reliable data on which to base management goals.

In general, national hunting trends are determined from hunter participation data (e.g., number of hunters, hunter recruitment, hunter retention, and number of days spent hunting [2–5]. In Hawai‘i, however, participation indicators alone are unlikely to accurately represent hunting trends for several reasons. First, hunter participation data alone tell us little about the impact that hunting has on wild populations of game species, and thus whether hunting plays a significant role in Hawaii’s fundamental need for game population control. In addition, the relationship between participation and game populations is unclear (i.e. do decreasing trends in hunter participation indicate fewer available game?). Second, unlicensed hunters are likely a significant portion of Hawaii’s hunters (perhaps as many as half of all hunters (Edwin Johnson, pers. comm.)), especially in recent years due to implementation of a hunter education program (Edwin Johnson, pers. comm.), making it difficult to discern whether declining trends in participation data are the result of actual declines in hunting activity or simply a decreasing portion of the population that purchases hunting licenses. Third, participation data do not take into account inactive hunters and hunter associates, resulting in inaccurate estimates of stakeholder numbers [5]. Game harvest data, on the other hand, add clarity to the question of hunter impact while lending clues to the magnitude of local game populations—two factors that are fundamental in determining management goals in Hawai‘i.

Few researchers have utilized state-reported data in analyses of historical hunting trends, citing the national FHWAR survey data as the most common primary resource [2–4]. To date, FHWAR data covers 13 surveys spanning 50 years (1955–2016). In Hawai‘i, FHWAR sample sizes are often very small (e.g., 10–29 individuals), and many of the results are not statistically robust [1]. Thus, the national survey data for Hawai‘i are unreliable and likely misrepresents the actual hunting trends in the state.

To date, the most comprehensive game harvest and hunter effort data in Hawai‘i have been collected primarily from self-reported hunter check-stations since the early 1940s. Since 1946, these check station data have been compiled into reports by the Hawai‘i State Division of Forestry and Wildlife (DOFAW) and submitted to the USFWS annually in order to qualify for P-R funds (DOFAW 2008, unpub. data). Due to the nature of self-reporting, there is potential for bias and inaccuracy in reported data. Nevertheless, these data provide the most complete hunting records available today for the state. For these reasons, state reported P-R program data provide more accurate estimates of hunter activity in Hawai‘i than FHWAR data.

Our goal was to understand historical hunting trends in the state of Hawai‘i in order to improve historical game harvest estimates. Based upon this goal, our objectives were to investigate the influence that time, location, and species have had on both game harvest and hunter participation from 1946 to 2008 using state-reported data. Since Hawai‘i is comprised of several geographically distinct islands with varying population densities and a range of socioeconomic and cultural conditions, we analyzed data at both state and island levels in order to discern whether trends varied locally or were consistent across the state. In addition, data were analyzed separately for all species, individual species, and taxonomic groups (mammals and birds) to determine whether species preference has changed over time. This study provides the first ever analysis of public hunting data in the state of Hawai‘i, and is one of only a handful to investigate long-term hunting dynamics in the US or around the world.

## Methods

**Fig 1:**
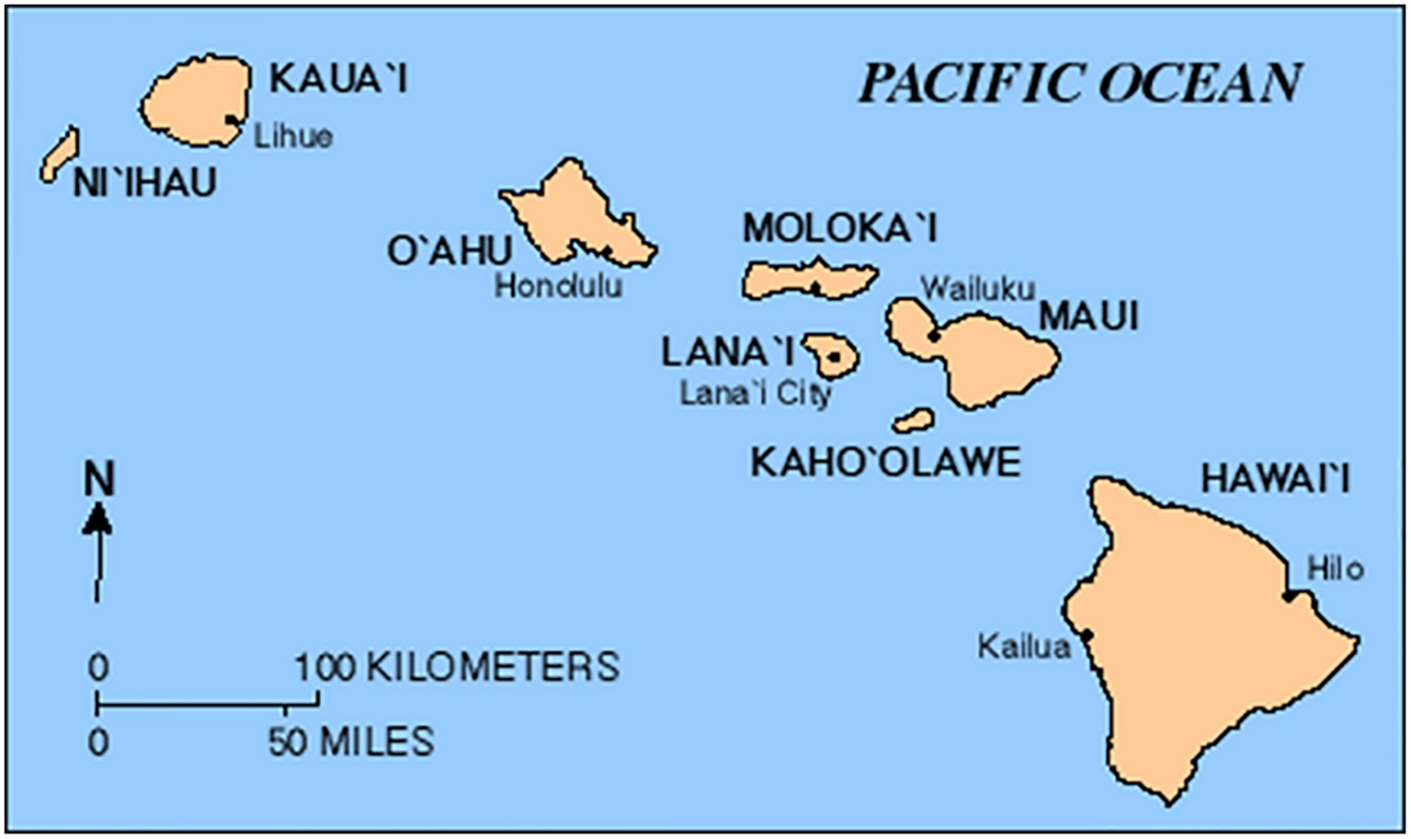
Map of the main Hawaiian Islands. Public domain image from the USGS.

A complete record of all hunting data from six Hawaiian Islands (Hawai‘i, Kaua‘i, Lāna‘i, Maui, Moloka‘i, O‘ahu; Fig 1) was obtained from DOFAW biologists in the form of hard copies of annual Pittman-Robertson reports (henceforth referred to as P-R reports) that provide estimates of state hunting data spanning 61 years (1946–2008). These records are the most complete document of hunting in the state and many exist only in the agency’s library.

Typically, P-R reports list data from individual game management areas, summarizing the annual number of animals harvested by island and species, number of hunters, and hunter hours for each year since 1946 (reports utilize federal fiscal calendar after 1953). For some years, P-R reports detailed population counts, eradication efforts, and the release of new game species. All relevant data were recorded from each available P-R report, though the format and exact content varied widely among reports. For instance, available P-R data for Kaua‘i in 1956 summarized annual harvest, number of hunters, hunter hours, and open days for ring-necked pheasants (*Phasianus colchicus*) only, whereas in 1957, Kaua‘i data were available for annual harvest and hunter trips for ring-necked pheasants, chukar (*Alectoris chukar*), and California quail (*Callipepla californica*), as well as goat population counts and estimates. Additional mammalian harvest and eradication data were also collected from available Reports of the Board of Commissioners of Agriculture and Forestry of the Territory of Hawai‘i from 1946–1959 (annual summaries covering a variety of topics that were prepared by the Division of Fish and Game for the Governor’s office). Although the majority of data from these two sources were consistent, in the rare cases where they overlapped in year and location, any discrepancies were settled in favor of P-R data. While some reports included population data (actual counts and estimates), hunter hours, hunter trips, hunter success rates (game/trip), the number of open days, and the number of permits issued, these data were relatively rare among reports and were sometimes reported multiple times for different parameters (e.g., in several instances, the same data were reported as both hunter hours and hunter trips). For these reasons, only the data that were consistently recorded were utilized for explanatory purposes in our analysis, these included year, species harvested, location, and hunter effort.

After all data were entered into a database they were edited as follows. First, if data were unavailable for a species for ≥25% of the years since it was introduced to Hawai‘i, the species was omitted from the analysis. The criteria for omission varied for each species since several species were introduced during the study period [11]. Omitted species included feral cattle (*Bos taurus*), mouflon-feral sheep hybrids (*Ovis musimon* × *O*. *aries*), pronghorn antelope (*Antilocarpa americana*), Northern bobwhite (*Colinus virginianus*), Chilean tinamou (*Nothoprocta perdicaria*), green pheasant (*Phasianus versicolor*), mourning dove (*Zenaida macroura*), common peafowl (*Pavo cristatus*), and Reeve’s pheasant (*Syrmaticus reevesii*). Second, species for which numbers were reported as composites with another species (e.g., mouflon sheep + mouflon-feral sheep hybrids, black francolin + gray francolin) as well as animals that were recorded under generic terminology that could apply to more than one species (i.e. “pigeons” and “quail”) were also omitted from the analysis. Third, often, a single P-R report listed data from multiple years, species, and/or locations, making the same data available in numerous reports. Where repeat data were found, some discrepancies existed among two or more reports. In these cases, the quantity reported the majority of the time was used. For example, two reports listed the number of bird hunters on the island of Hawai‘i in 1959 as 1,485, while another report listed the same case as 1,336—in such an instance, 1,485 was used and 1,336 was excluded. When only two conflicting values were found for the same instance, the mean of the two values was used. Fourth, when data were presented as a range of values rather than as a single number, an average of the range was used. Fifth, in occasional instances, the number of animals harvested was not explicitly mentioned in any report. In these instances, it was possible to calculate harvest from other data provided. For example, hunter success (the number of animals harvested per number of hunters) was multiplied by the number of hunters to determine harvest for a given species. In most cases, data from individual game management areas were added together to determine island totals, species totals, and statewide totals, though a few reports listed sum totals for various years or locations. Results from state eradication efforts were not included as public hunting data.

### Statistical analysis

#### Statewide game harvest

We developed nine *a priori* models to investigate statewide trends in game harvest from 1946 to 2008 by utilizing the sum of animals harvested annually for all hunting areas on all islands, where data were recorded as the dependent variable and time (year) and taxonomic grouping (birds or mammals) as the explanatory variables. The first four models examined the effect of time while controlling for taxonomic group for the entire sample (models 1–4, Table 2), while the remaining five models were run using only bird or mammal data in order to investigate trends and significance of each group separately (models 5–9, Table 2).

**Table 2:** Models used to test statewide and islandwide game harvest and hunter participation in Hawai‘i between 1946 and 2008.

Model	Description
0	null (constant only)
1	harvest or hunters = constant + year + taxon
2	harvest = constant + year + year^2^ + taxon
3	harvest = constant + year + year^2^ + year^3^ + taxon
4	harvest or hunters = constant + year + taxon + year × taxon
5	harvest = constant + year
6	harvest = constant + year + year^2^
7	harvest = constant + year + year^2^ +year^3^
8	harvest = constant + year + island
9	harvest = constant + year + island × year
10	hunters = constant + year
11	hunters = constant + year + year^2^
12	hunters = constant + year + year^2^ + year^3^
13	hunters = constant + year + island
14	hunters = constant + year + island × year

#### Game harvest by island

Historical trends in game harvest on individual islands were analyzed using nine models for each of the six islands (54 total models). Specifically, three models (models 5–7, Table 2) each for birds, mammals, and total animals harvested, respectively, were run for each island, in which harvest was the dependent variable and time (years) was the independent variable. Total animals harvested on each island (the sum of bird and mammal totals) were analyzed using only the years in which data were available for both birds and mammals, resulting in the omission of 13 years of data on Hawai‘i, 13 years on Kaua‘i, 37 years on Lāna‘i, 42 years on Maui, 40 years on Moloka‘i, and 35 years on O‘ahu.

#### Individual species harvested across the islands

Sufficient data were available to investigate islandwide trends for 19 individual species (6 mammals and 13 birds). For each island, a species was included in the analysis only when data were available for ≥25% of the years since the original introduction. Omissions from analysis included gray francolin (*Francolinus pondicerianus*) on Kaua‘i; goats (*Capra hircus*), Erckel’s francolin (*Francolinus erckelii*), and Japanese quail (*Coturnix japonica*) were excluded on Lāna‘i; axis deer (*Axis axis*), black francolin (*Francolinus francolinus*), gray francolin, Japanese quail, spotted dove (*Streptopelia chinensis*), and zebra dove (*Geopelia striata*) were excluded on Maui; axis deer, chukar (*Alectoris chukar*), Gambel’s quail (*Callipepla gambelii*), Japanese quail, ring-neck pheasants (*Phasianus colchicus*), spotted dove, turkey (*Meleagris gallopavo*), and zebra dove were excluded on Moloka‘i; and black francolin, chukar, gray francolin, and Japanese quail were excluded from analyses on O‘ahu. A simple linear model (model 5, Table 2) was run for each species for which data were available from only one island (or where island was not an important categorical variable), which included feral sheep (*Ovis aries*), Kalij pheasant (*Lophura leucomelanos*), and Chestnut-bellied sandgrouse (*Pterocles exustus*) on Hawai‘i, black-tailed deer (*Odocoileus hemionus columbianus*) on Kaua‘i, axis deer on Lāna‘i, and Gambel’s quail and mouflon sheep on Hawai‘i and Lāna‘i. For the remaining 12 species, annual harvest trends were explained using linear models that included both time (year) and location (island) (models 8 and 9, Table 2).

#### Hunter participation

We developed a total of seven models to investigate hunter participation. The first set of models followed harvest models 1 and 4 and explained the annual number of hunters using time, taxonomic group, and their interaction as independent variables (models 1 and 4, Table 2). To further investigate this relationship, a second set of models were run (models 10–12, Table 2) for each of three groups: 1) all hunters (birds and mammals) reported in the state; 2) those hunting birds; and, 3) those hunting mammals (9 models). The final set of models examined hunter participation over time across different islands by utilizing time and dummy variables for each island as explanatory variables (models 13 and 14, Table 2). Data were considered acceptable for analysis for an island only when data were available for ≥ 25% of the study period (i.e. ≥ 15 years).

#### Model selection

Temporal trends in game harvest and hunter participation were analyzed using a regression framework. Both linear and nonlinear models were explored, and several models employed quadratic or interaction terms (see Table 2 for models). We also included null models for model comparisons. We selected best-fit models within each model grouping based on Akaike information criteria corrected for sample size (AICc), with models exhibiting ΔAICc ≤ 2.0 from the best-fit model considered as equivalent [12]. In cases where two or more models were equally weighted, the simpler model was chosen as AIC can overparameterize models by adding variables that are not significant and do not improve fit [13]. Notably, no model comparisons were run for the five species that had harvest data for only one island. Outliers were examined on a case by case basis in order to determine whether or not to omit them from the analysis. Extreme values that could be explained (e.g., large drives to reduce game populations, the opening of a new hunting area or new species) or that were consistent with other parameters for that year (i.e. number of hunters, hunter hours, or hunter trips) were kept and all others were excluded. Ultimately, 7 outliers were kept and 6 outliers were excluded from the analysis. Of the 6 exclusions, 4 were supplemental data from Reports of the Board of Commissioners of Agriculture and Forestry of the Territory of Hawai‘i from 1946–1959 that reported public hunt results and state-led eradication numbers together, and 2 were very dissimilar to other data from the same year and surrounding years. All statistical analyses were conducted using Systat 13.

## Results

### Statewide game harvest

**Fig 2:**
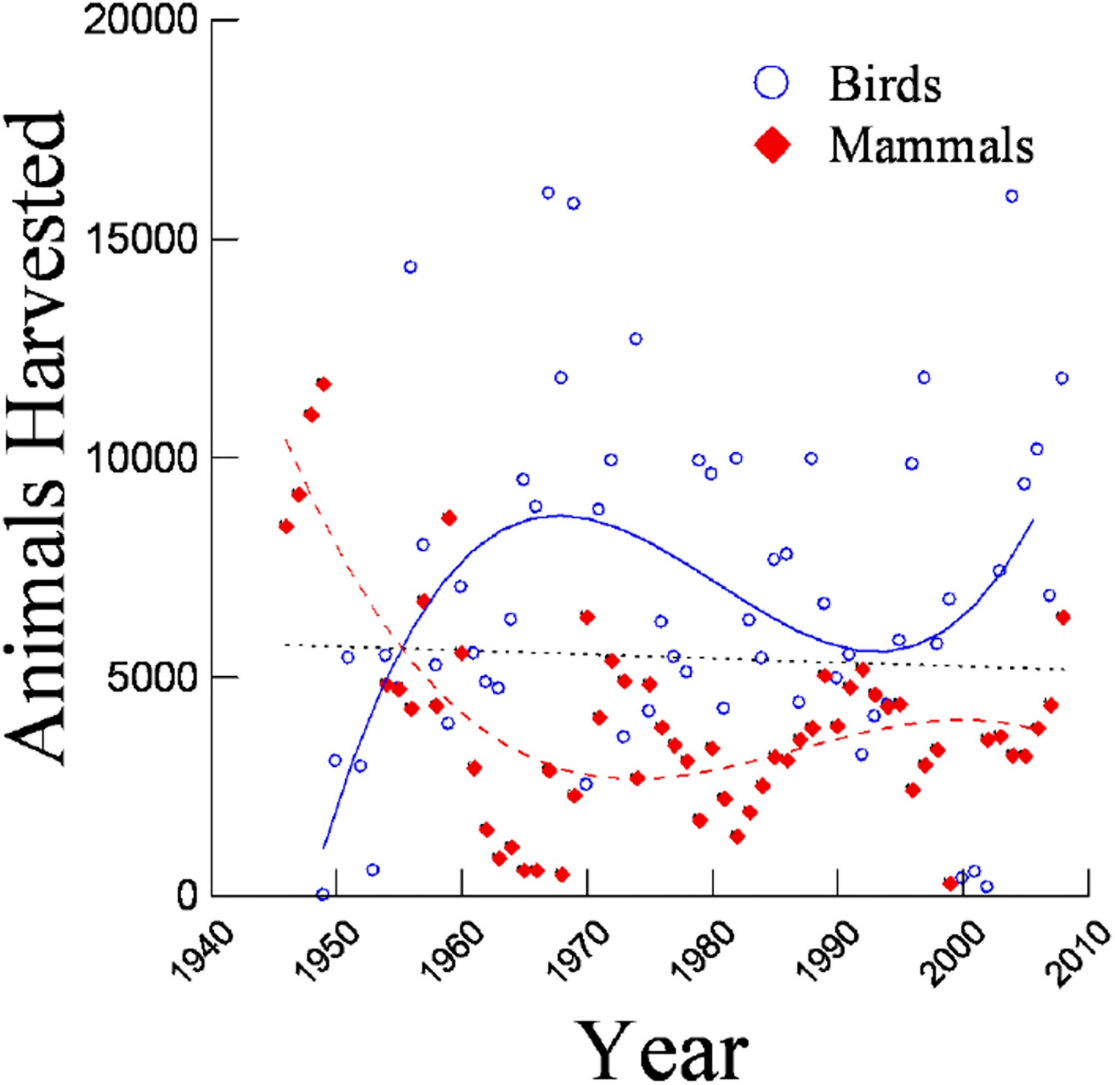
Trends in statewide harvest of all game species (dotted line), birds (solid line), and mammals (dashed line) in Hawai‘i between 1946 and 2008.

The best-fit model for total animals harvested annually across Hawai‘i was model 4 harvest = constant + year + taxon + year × taxon) which indicated that birds and mammals showed different temporal trends (Table 3). When considered separately, models indicated that bird and mammal harvest did show a marked change over time (Table 4). The best-fit models for the bird and mammal harvests indicated that for both taxa, the cubic model was the most heavily weighted (Table 4) with inflections occurring around the late 1960s, though each showed opposite trends in statewide harvest over time (Fig 2).

**Table 3:** Statewide total game harvest results for models 0–4* (n = 117).

		Coefficients								
Model	df	Constant	Year	Year^2^	Year^3^	Taxon	Interaction	R^2^	AICc	ΔAICc
0		5487.3						0.0	2242.6	19.6
1	2	30186.8	-13.5			1375.0		0.161	2226.3	3.3
2	3	5283633.0	-5324.5	1.3		1374.6		0.173	2227.4	4.4
3	4	-3.6 × 10^8^	553770.2	-281.4	0.05	1366.7		0.177	2228.3	5.3
4	3	32217.4	-13.5			-75817.4	39.0	0.200	2223.0	0

*see Table 2 for model descriptions.

**Table 4:** Statewide bird (n = 60) and mammal (n = 57) harvest results for models 5–7*.

			Coefficients							
	Model	df	Constant	Year	Year^2^	Year^3^	Adj. R^2^	*p*	AICc	ΔAICc
Birds	0		6823.8						1163.5	6.8
	5	1	-43600.0	25.5			0.000	0.376	1164.9	8.2
	6	2	-8.6 × 10^6^	8689.8	-2.2		0.004	0.337	1165.7	9.0
	7	3	-3.0 × 10^9^	4.5 × 10^6^	-2262.2	0.4	0.162	0.005	1156.7	0
Mammals	0		4080.4						1050.3	32.7
	5	1	1.1 × 10^5^	-52.6			0.151	0.003	1043.2	25.6
	6	2	1.7 × 10^7^	-16824.9	4.2		0.433	<0.001	1022.5	4.9
	7	3	1.0 × 10^9^	-1.5 × 10^6^	764.6	-0.1	0.506	<0.001	1017.6	0

*see Table 2 for model descriptions.

### Game harvest by island

**Fig 3:**
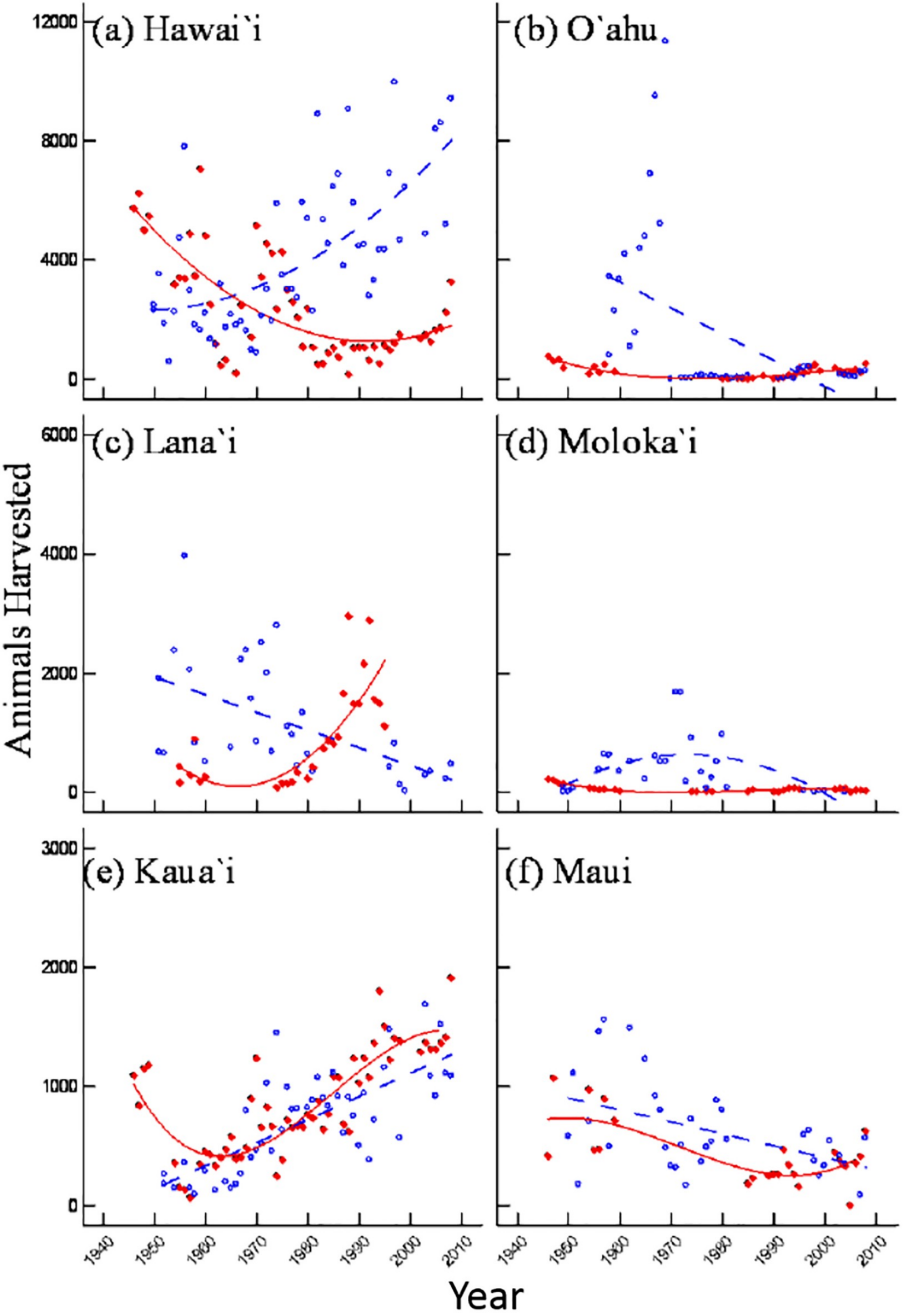
Trends in statewide harvest of birds (solid line) and mammals (dashed line) on six of the main Hawaiian Islands between 1946 and 2008.

Historical harvest trends varied among the six islands. Over the 61 years the number of game birds and mammals harvested decreased on Maui and O‘ahu but increased on Kaua‘i (Fig 3). On Hawai‘i, total animal harvest followed a positive quadratic pattern, showing a decrease around the 1970s. In general, game harvest was greatest on Hawai‘i, with up to 12,000 animals killed each year. Overall harvest was lowest on Kaua‘i and Maui with reported kills numbering consistently below 2,000 birds or mammals annually (Fig 3). Finally, across the six islands the best models for total animals harvested were either quadratic (model 6) or cubic (model 7, Table 5).

**Table 5:** Islandwide game harvest results for models 5–7*.

Parameters (n)^a^	Model	Adj. R^2^	df	*p*	AICc	ΔAICc
H, A (61)	0				1147.6	10
	5	0.157	1	<0.001	1138.4	0.8
	6	0.186	2	<0.001	1137.6	0
	7	0.179	3	0.003	1139.4	1.8
H, B (57)	0				1070.1	22.8
	5	0.343	1	<0.001	1047.4	0.1
	6	0.358	2	<0.001	1047.3	0
	7	0.355	3	<0.001	1049.0	1.7
H, M (54)	0				962.9	34.2
	5	0.369	1	<0.001	939.2	10.5
	6	0.493	2	<0.001	928.7	0
	7	0.491	3	<0.001	930.3	1.6
K, A (57)	0				924.2	65.5
	5	0.674	1	<0.001	861.6	2.9
	6	0.679	2	<0.001	862.0	3.3
	7	0.703	3	<0.001	858.7	0
K, B (47)	0				703.9	26.0
	5	0.440	1	<0.001	677.9	0
	6	0.438	2	<0.001	679.4	1.5
	7	0.437	3	<0.001	680.9	3.0
K, M (55)	0				834.9	54.5
	5	0.441	1	<0.001	804.2	23.8
	6	0.561	2	<0.001	792.2	11.8
	7	0.654	3	<0.001	780.4	0
L, A (47)	0				778.4	3.0
	5	0.072	1	0.038	776.1	0.7
	6	0.112	2	0.027	775.4	0
	7	0.096	3	0.062	777.6	2.2
L, B (31)	0				517.8	9.2
	5	0.291	1	0.001	508.6	0
	6	0.281	2	0.004	510.6	2.0
	7	0.320	3	0.004	510.6	2.0
L, M (26)	0				427.7	19.2
	5	0.403	1	<0.001	415.8	7.3
	6	0.577	2	<0.001	408.5	0
	7	0.608	3	<0.001	408.5	0
MA, A (51)	0				834.0	15.8
	5	0.218	1	<0.001	822.7	4.5
	6	0.302	2	<0.001	818.2	0
	7	0.289	3	<0.001	820.5	2.3
MA, B (33)	0				488.9	6.3
	5	0.207	1	0.005	482.6	0
	6	0.181	2	0.019	485.2	2.6
	7	0.171	3	0.038	487.3	4.7
MA, M (25)	0				422.6	6.9
	5	0.270	1	0.005	416.2	0.5
	6	0.335	2	0.004	415.7	0
	7	0.326	3	0.010	418.0	2.3
MO, A (53)	0				855.3	14.5
	5	0.084	1	0.020	851.9	11.1
	6	0.202	2	0.001	845.8	5.0
	7	0.293	3	<0.001	840.8	0
MO, B (29)	0				481.1	7.1
	5	0.035	1	0.166	481.5	7.5
	6	0.296	2	0.004	474.0	0
	7	0.320	3	0.005	474.8	0.8
MO, M (32)	0				396.9	52.8
	5	0.261	1	0.002	388.6	44.5
	6	0.562	2	<0.001	373.4	29.3
	7	0.834	3	<0.001	344.1	0
O, A (54)	0				995.6	9.1
	5	0.064	1	0.036	993.2	6.7
	6	0.101	2	0.066	994.4	7.9
	7	0.212	3	0.002	986.5	0
O, B (40)	0				749.5	8.7
	5	0.221	1	0.001	740.8	0.0
	6	0.248	2	0.002	740.8	0.0
	7	0.268	3	0.003	741.3	0.5
O, M (33)	0				449.8	33.9
	5	0.124	1	0.025	446.8	30.9
	6	0.671	2	<0.001	416.0	0.1
	7	0.688	3	<0.001	415.9	0

*see Table 2 for model descriptions.

^a^H = Hawai‘i Island, K = Kaua‘i, L = Lāna‘i, MA = Maui, MO = Moloka‘i, O = O‘ahu; A = all species, B = birds, M = mammals

When analyzed separately, harvest of the two taxonomic groups varied independent of game harvest totals. The number of game birds harvested on Hawai‘i and Kaua‘i increased by approximately 300%, while game bird harvest decreased on Lāna‘i and Maui by a similar amount. Bird harvest followed a decreasing trend on O‘ahu as well, with reported numbers falling from 5,000–10,000 annual kills during the 1960s to a few hundred or less from the 1970s on. On Moloka‘i, game bird harvest peaked in the 1970s and has been decreasing ever since. The best-fit models for game bird harvest indicate that change was linear (model 5) on half the islands and quadratic on the other half (model 6, Table 5). Mammal harvest, on the other hand, showed a downward trend on all islands except Kaua‘i and Lāna‘i (Fig 3). Across the islands the best-fit models indicated that changes in game mammal harvest exhibited primarily either a quadratic (model 6) or cubic relationship (model 7, Table 5) in which the inflection occurred approximately between 1970 and 1990 (Fig 3).

### Individual species harvested across the islands

Variation among historical harvest trends for 19 species were best explained using three models (Tables 6–8). Simple linear models were used for the seven single-island species (or where island was not a factor) (Table 6) and multiple regression models were used for the remaining 12 species—eight models resulted in a significant interaction between time and location (Table 7), and four models included time and location (without interaction) (Table 8). In two cases the best models did not meet the criteria of having any significant relationship for the species being considered. Specifically, the best fit models for Chestnut-bellied sandgrouse and Gambel’s quail were not significant (Table 6).

**Table 6:** Islandwide game harvest models of single species occurring on a single island or where islands were not different (model 10; Harvest = constant + year).

					Coefficients (*p*)		
Species	Island^a^	n	Adj. R^2^	df	Constant	Year	*p*
Axis deer	L	23	0.375	1	-79472.1 (0.001)	40.6 (0.001)	0.001
Black-tailed deer	K	36	0.695	1	-2495.8 (<0.001)	1.3 (<0.001)	<0.001
Feral sheep	H	39	0.398	1	76912.1 (<0.001)	-38.3 (<0.001)	<0.001
Mouflon sheep	H, L	47	0.304	1	-38308.0 (<0.001)	19.4 (<0.001)	<0.001
Gambel’s quail	H, L	40	0.032	1	-2326.2 (0.147)	1.2 (0.139)	0.139
Kalij pheasant	H	30	0.203	1	-10610.3 (0.008)	5.4 (0.007)	0.007
Sandgrouse	H	13	0.000	1	335.1 (0.622)	-0.2 (0.629)	0.629

^a^Refers to islands that were included in the analysis; H = Hawai‘i Island, K = Kaua‘i, L = Lāna‘i, MA = Maui, MO = Moloka‘i, O = O‘ahu

**Table 7:** Islandwide game harvest models of species occurring on more than one island in which the best models showed an interaction between island and time (model 14; Harvest = constant + year + island + island × year). Note that only the interaction terms are displayed.

					Coefficients						
Species	Islands^a^	n	R^2^	df	Constant	H×Yr	K×Yr	L×Yr	MA×Yr	MO×Yr	*p*
Goat	H, K, MA, MO, O	171	0.404	9	14216.0	-2.4	12.7		-18.2	-2.8	<0.001
Pig	H, K, MA, MO, O	171	0.820	9	14680.0	-35.9	15.1		7.7	8.0	<0.001
Zebra dove	H, K, L, O	148	0.427	7	29366.6	17.1	20.8	3.8			<0.001
California quail	H, K, MA, MO	132	0.604	7	-8,479.8	22.9	-5.1		-7.0		<0.001
Erckel’s francolin	H, K, O	90	0.733	5	-45052.4	39.2	-16.0				<0.001
Spotted dove	H, K, L, O	146	0.374	7	23631.0	13.6	15.3	-2.1			<0.001
Ring-necked pheasant	H, K, L, MA, O	185	0.501	9	20239.0	-15.1	8.4	-2.1	-0.1		<0.001
Gray francolin	H, L, MO	80	0.430	5	15133.8	9.6		-5.6			<0.001

^a^Refers to islands that were included in the analysis; H = Hawai‘i Island, K = Kaua‘i, L = Lāna‘i, MA = Maui, MO = Moloka‘i, O = O‘ahu

**Table 8:** Islandwide game harvest models of species occurring on more than one island in which the best model showed no interaction (model 13; Harvest = constant + year + island).

					Coefficients					
Species	Islands^a^	n	R^2^	df	Constant	Year	Hawai‘i	Kaua‘i	Lāna‘i	*p*
Black francolin	H, K, MO	65	0.391	3	-17996.0	9.1	298.3	-16.6		<0.001
Chukar	H, K, L, MA	136	0.584	4	-2598.4	1.5	725.3	-343.0	-259.4	<0.001*
Japanese quail	H, K	86	0.236	2	-1591.1	0.9	82.2			0.001*
Turkey	H, L	50	0.368	2	-6464.4	3.3	86.3			<0.001

^a^Refers to islands that were included in the analysis; H = Hawai‘i Island, K = Kaua‘i, L = Lāna‘i, MA = Maui, MO = Moloka‘i, O = O‘ahu

*Islands differed, but year was not significant.

**Fig 4:**
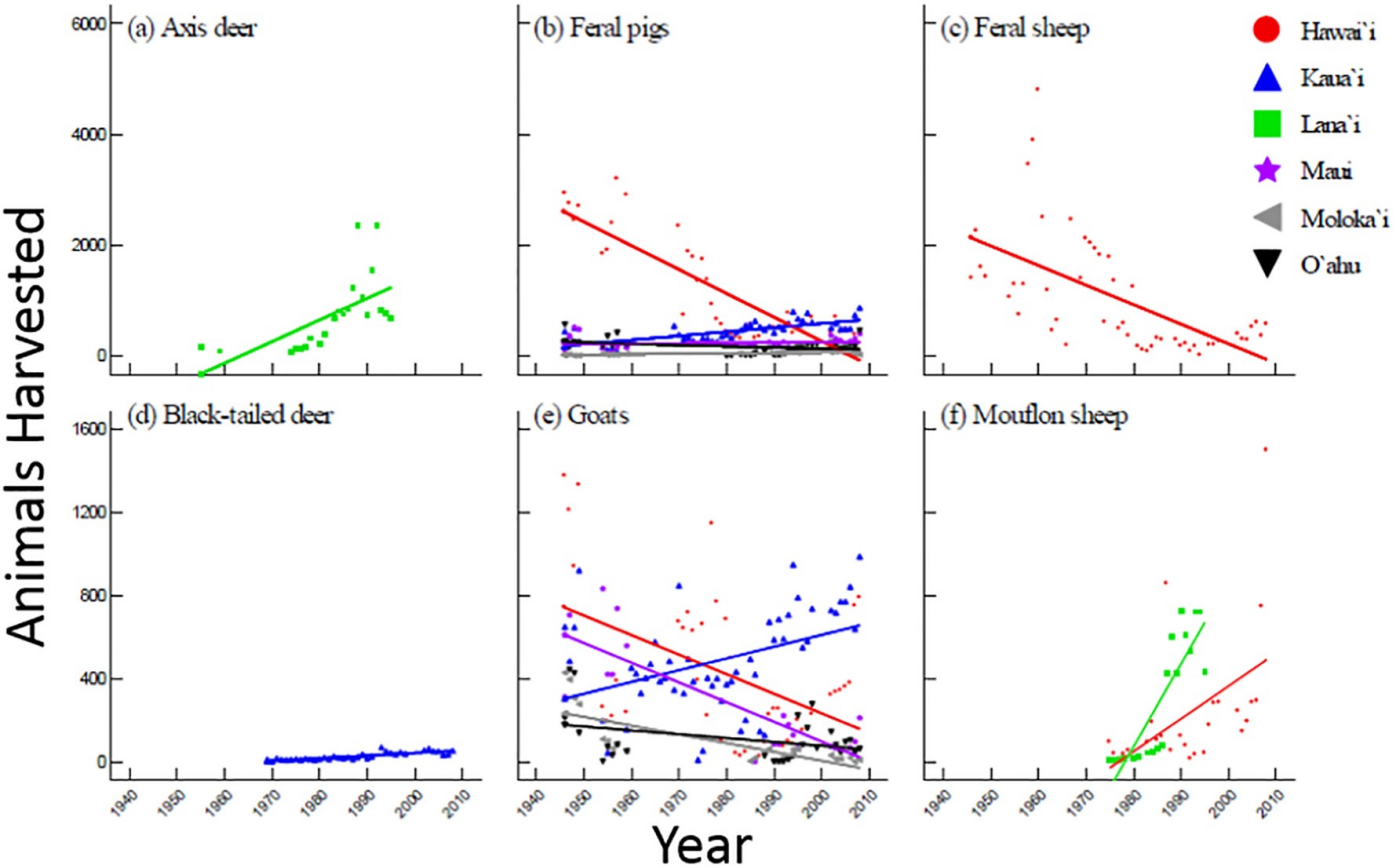
Trends in islandwide harvest of game mammals in Hawai‘i between 1946 and 2008.

**Fig 5:**
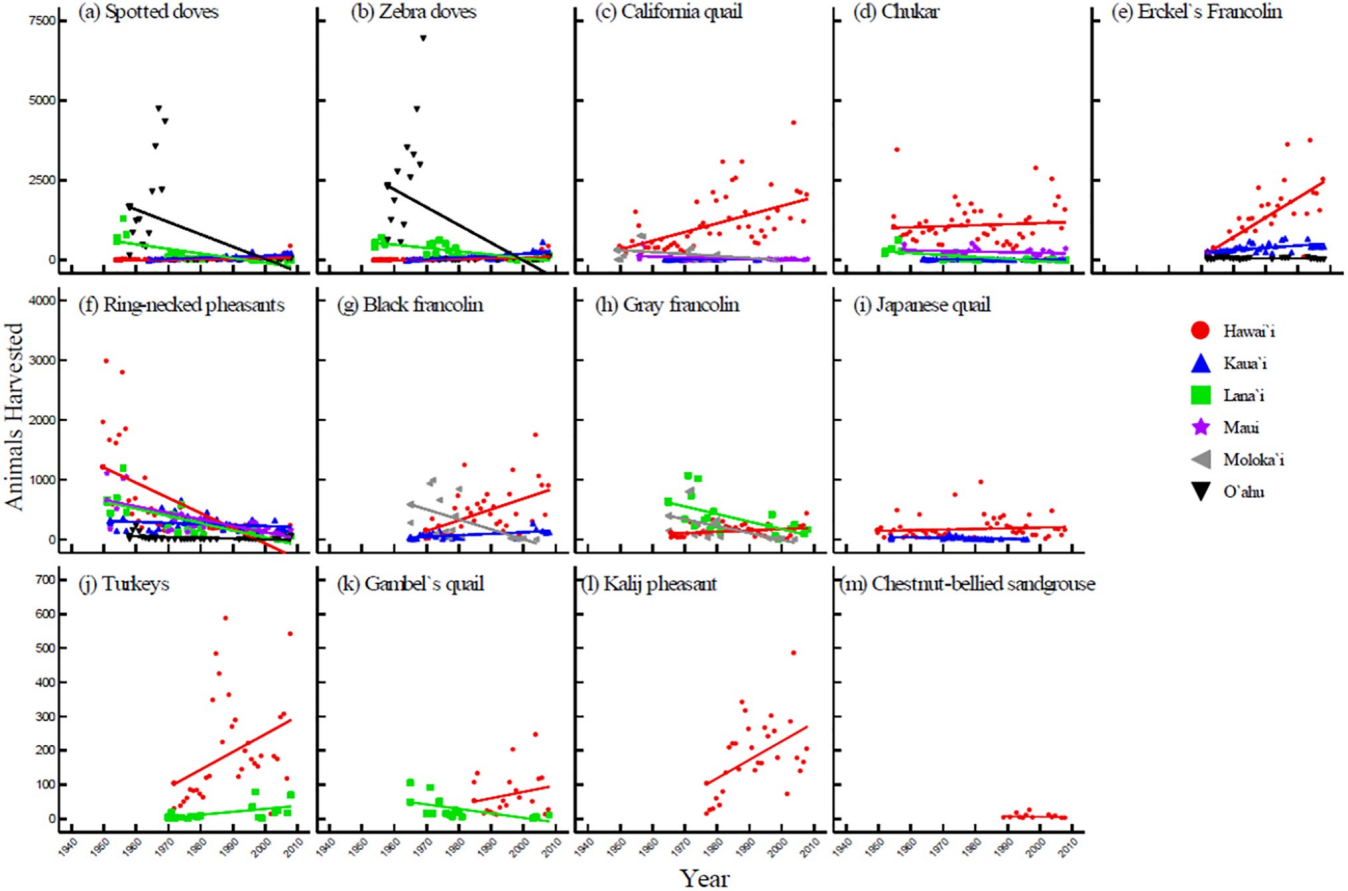
Trends in islandwide harvest of game birds in Hawai‘i between 1946 and 2008.

Game harvest was inconsistent among islands for each species (Figs 4 and 5). In general, results were consistent with the islandwide trends found for total birds and total mammals (Fig 3; Table 5), though some species deviated from these patterns. Overall, mammal harvest showed an upward trend on Kaua‘i and Lāna‘i and a downward trend on Hawai‘i for all species except mouflon sheep (Fig 4, Tables 6–8). Bird harvest increased over time on Hawai‘i for all species except ring-necked pheasants (Fig 5). Similarly, bird harvest on Kaua‘i consistently stayed the same or increased over time for all species except ring-necked pheasant and chukar (Fig 5). On Maui and Moloka‘i, bird and mammal harvests were either stable or decreasing for all species (Figs 4 and 5).

### Hunter participation

**Fig 6:**
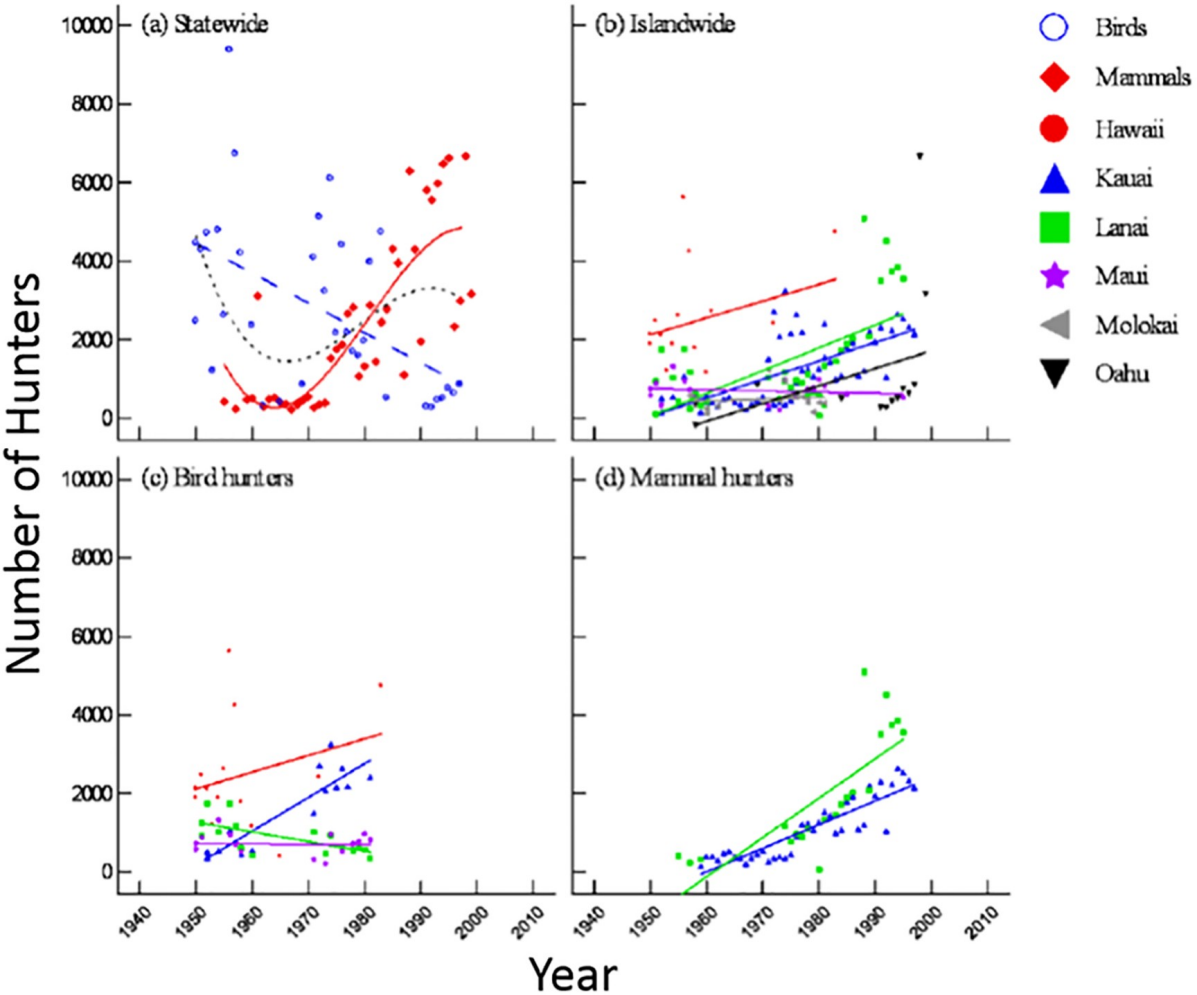
Hunter participation results for (a) statewide total (dotted line), bird (dashed line), and mammal (solid line), (b) all species by island, (c) birds by island, (d) mammals by island.

Historical trends in hunter participation varied according to time, taxonomic group, and island (Fig 6, Table 9). In the first set of models, there was greater support for the model that tested an interaction between time and taxonomic group than the model that did not include an interaction term (ΔAIC = 40.2; Table 9a). A closer look revealed that the number of statewide hunters decreased linearly for birds but increased sigmoidally for mammals (Fig 6a, Table 9b).

**Table 9:** Trends in hunter participation explained by (a) time and taxonomic group, (b) models 10–12*, and (c) different islands.

a)			Coefficients (*p*)										
n	R^2^	df	Constant	Year	Taxon	Yr×Tax	AICc	ΔAICc					
76	0.029	2	-42054.6	22.6 (0.220)	261.1 (0.307)		1387.9	40.2					
76	0.446	3	-49130.3	26.0 (0.065)	201969.4 (<0.001)	-102.1 (<0.001)	1347.5	0					
b)					Coefficients (*p*)								
Parameter	Model	n	Adj. R^2^	df	Constant	Year	Year^2^	Year^3^	AICc	ΔAICc			
All species	0								1385.7	3.1			
	10	76	0.002	1	-35279 (0.322)	19.1 (0.290)			1386.7	4.1			
	11	76	0.046	2	1.1 × 10^7^ (0.040)	-11072.3 (0.039)	2.8 (0.039)		1384.5	1.9			
	12	76	0.084	3	1.7 × 10^9^ (0.047)	-2620793 (0.047)	1324.3 (0.097)	-0.2 (0.048)	1382.6	0			
Birds	0								606.1	7.5			
	10	33	0.237	1	1.52839.0 (0.002)	-76.1 (0.002)			598.6	0			
	11	33	0.238	2	-6867275 (0.323)	7039.9 (0.318)	-1.8 (0.313)		600.1	1.5			
	12	33	0.213	3	2.6 × 10^8^ (0.852)	-391736.1 (0.850)	200.2 (0.849)	-0.03 (0.848)	602.8	4.2			
Mammals	0								783.3	39.1			
	10	43	0.587	1	-251099.7 (<0.001)	128.1 (<0.001)			746.6	2.4			
	11	43	0.605	2	9091464 (0.104)	-9320.0 (0.010)	2.4 (0.095)		746	1.8			
	12	43	0.634	3	1.8 × 10^9^ (0.048)	-2775838 (0.048)	1401.5 (0.049)	-0.2 (0.049)	744.2	0			
c)						Coefficients (*p*)							
Parameter	Model	Islands^a^	n	df	R^2^	Constant	Year	Island	H×Yr	K×Yr	L×Yr	AICc	ΔAICc
Birds	13	H, K, L, MA	56	4	0.425	-31416.3	16.7 (0.149)	(<0.0001)				928.5	7.9
	14	H, K, L, MA	56	7	0.568	-49013.9	25.7 (0.019)	(0.003)	16.8 (0.003)	60.1 (0.003)	-50.1 (0.003)	920.6	0
Mammals	13	K, L	60	2	0.679	-145991.7	74.5 (<0.001)	(<0.001)				955.1	4.8
	14	K, L	60	3	0.716	-157178.8	80.2 (<0.001)	(0.010)		-20 (0.010)		950.3	0

*See Table 2 for model descriptions.

^a^Refers to islands that were included in the analysis; H = Hawai‘i Island, K = Kaua‘i, L = Lāna‘i, MA = Maui, MO = Moloka‘i, O = O‘ahu

Islandwide analysis revealed that historical trends in hunter participation varied across islands. When analyzed together, bird and mammal hunters increased on all islands except Maui and Moloka‘i, where numbers remained relatively stable throughout the study period (Fig 6b). Taken separately, the amount of available bird hunter data were sufficient (available for ≥ 20% of the study period) for only Hawai‘i, Kaua‘i, Lāna‘i, and Maui between 1950 and 1980, and mammal hunter data were sufficiently available for only Kaua‘i and Lāna‘i from approximately 1955–2000 (Table 9c). Both bird and mammal hunters increased on Kaua‘i, whereas on Lāna‘i the number of mammal hunters increased but bird hunters decreased (Fig 6, Table 9c).

## Discussion

Since World War II, trends in game harvest and hunter participation in Hawai‘i have varied widely among islands and species. A comprehensive look at game harvest and hunter participation data for the entire state does not indicate simple, overarching historical hunting trends in Hawai‘i. In fact, statewide harvest and hunter data for all species combined show little to no correlation when analyzed as a whole (Fig 2; Table 9). When taxonomic group, island, and species data were viewed separately, however, clear trends emerge.

Changes in bird and mammal harvest often followed diverse patterns. In fact, changes in statewide bird and mammal harvest mirrored each other throughout the study period (Fig 2). These results appear to be correlated with changes in game species management [11]. Total bird harvest was generally higher than total mammal harvest, except immediately following WWII when mammals were first added to the official game list [11]. Mammal harvest declined until the 1950s and 1960s, when some small populations of trophy mammals were introduced to augment the existing mammalian game which consisted mostly of feral domesticates [11]. Since 1970, statewide mammal harvest has fluctuated, but generally remained at <7,000 individuals harvested per year. Bird harvest, on the other hand, rose steadily following WWII, peaking around the late 1960s when the number of game bird introductions also peaked (Fig 2, [11]). Game bird harvest declined during the 1970s and 1980s, which follows the end of game bird introductions in Hawai‘i. These results are consistent with FHWAR data for the United States as a whole which indicate that recreational hunting experienced a boom during the 1950s with numbers continuing to increase nationally until the mid-1970s. This boom is often attributed to the revival of the US economy following WWII which led to increased disposable income, increased leisure time, institutionalization of paid vacations, and improvements in personal transportation [3, 14]. Trends in overall hunting activity began to decline in the mid-1970s [3–4, 15] possibly due to the increasing urbanization and suburbanization of American society [2, 16], lack of access to suitable wilderness for hunting [16], an increase in non-consumptive recreational activities [16], and shifts in public acceptance of trophy versus subsistence hunting [16–18].

Game harvest trends deviated even further when bird and mammal data were divided among islands (Fig 3). Game harvest numbers were greatest on the island of Hawai‘i, the largest island in the state with the greatest number of game species (15 out of 15 bird species (data for 13 species were analyzed in this study) and 5 out of 7 mammal species (4 species analyzed) inhabit the island; Table 1). Unlike statewide trends, bird harvest increased on Hawai‘i throughout the study period, whereas mammal harvest decreased over time. Kaua‘i was the only other island where bird harvest increased during the study period (Fig 3). Similarly, mammal harvest on Kaua‘i increased following an initial decline from post-WWII numbers, making Kaua‘i the only island where both bird and mammal harvest showed increasing trends for most of the study period. Like Kaua‘i, mammal harvest increased on Lāna‘i following the 1960s, though mammal data for Lāna‘i are not available for recent years. The increasing trends on these two islands may be explained by the species present—both islands have only a fraction of the state’s seven game mammal species, but, with the exception of goats and pigs on Kaua‘i, they are relatively rare trophy species (i.e. black-tailed deer on Kaua‘i and mouflon sheep and axis deer on Lāna‘i). While axis deer are also present on Maui and Moloka‘i, they were not included in the analysis for those islands due to the lack of data. Although species composition may contribute to the diverse game harvest patterns observed among islands, it is important to consider that other factors such as terrain, quality and quantity of hunting areas, and socioeconomic climate differ among islands.

Harvest of individual species correlated with island trends in most cases, though there were a few exceptions (Figs 4 and 5). On the island of Hawai‘i, pig, feral sheep, and goat harvest declined while mouflon sheep harvest increased. The rise in mouflon kills during the 1980s and 1990s was a result of state government efforts to increase public hunting through easing of hunting restrictions combined with aerial shooting of the species by agency personnel in order to comply with court-ordered eradication efforts on Mauna Kea [11, 19]. Among game birds, trends in ring-necked pheasant harvest were the most notable since harvest of the species declined across the state regardless of local bird harvest trends. On O‘ahu, the steeply declining trend in game bird harvest was strongly influenced by the high numbers of spotted doves and zebra doves harvested during the 1950s and 1960s. For both species of dove, harvest numbers dropped abruptly from upwards of 4,000 birds per year during the 1960s to less than 5 birds during the early 1970s, presumably due to overhunting.

Statewide hunter participants also diverged according to taxonomic group, though hunter participation trends were not correlated with harvest trends in most cases. The number of statewide mammal hunters increased from less than 500 in the 1960s to several thousand during the 1990s (Fig 6a), yet harvest data indicate that the number of mammals harvested in Hawai‘i remained relatively stable during that period (Fig 2). Similarly, bird hunters declined linearly (Fig 6a), but birds harvested followed a sigmoidal pattern during the study period (Fig 2). These results suggest a few possibilities: the number of animals actually harvested per hunter changed over time; the number of mammals reported per hunter changed; or the number of hunters that reported their kills changed at a different rate than the rate of actual kills. Interestingly, statewide hunter participation rates most closely resemble harvest rates during the 1970s and 1980s, diverging again during the 1990s (Figs 2 and 6a). These changes are consistent with patterns of resource management in the state in which the 1960s and 1970s marked the time when Hawaii’s hunting program was arguably the most organized and closely monitored. Since the 1970s, resource management priorities have been shifting away from extractive uses to more non-consumptive activities centered on conservation and restoration [11], though the vast majority of areas open to hunting have continued to be hunting focused, not conservation focused. In particular, the island of Kaua‘i has had virtually zero conservation actions taken (until recently) that would negatively impact game populations.

In some cases, Hawaiian trends follow a similar pattern to national trends, yet most of the data do not. Similar to FHWAR findings, statewide bird harvest did increase until the 1970s and then decrease thereafter (Fig 2). However, Hawaiian bird harvest began to rise again around the 1990s—a change that neither occurred nationally nor was anticipated by earlier research. Statewide mammal harvest, on the other hand, dropped slightly during the 1970s and has remained relatively constant since (Fig 2). Harvest trends at the island and species level were much more variable, though much of the data with a quadratic or sigmoid pattern did show an inflection around the 1970s (Figs 3 and 4). The causes behind the wide range of variability in data from Hawai‘i relative to national trends cannot be determined conclusively from harvest data alone, though it is possible that the low number of hunters in Hawai‘i when compared with mainland states is a contributing factor.

Historical analysis of Hawaii’s hunting data is complicated by the fact that game species were being introduced until about 50 years ago and their populations may still be expanding, similar to the increase in number of introduced passerine birds in the state. In addition, the interplay between introduced game and sensitive island ecosystems makes Hawaii’s hunting program exceptional among US states [5]. Although P-R reports provide the most complete picture available today of historical game harvest in Hawai‘i, these data are likely biased. Sources of bias in Hawaiian hunters have not been determined conclusively, but it is probable that an underrepresentation of game harvest at self-check stations is the result of a desire to keep good hunting territory secret from other hunters or government conservation (i.e. eradication) efforts (Ed Johnson pers. comm.). Furthermore, existing P-R data are incomplete and may not accurately represent exact quantities of game harvested or hunters going afield. In fact, the exact quantities reported here are likely underestimates of actual historical numbers due to reporting errors, poaching, or loss of data through the years. However, these data are adequate in order to achieve the primary objective of this research, which was to interpret generalized temporal trends to obtain a big-picture perspective on both changes in the rate of removal of introduced game and changes in levels of hunter participation in Hawai‘i since WWII. Furthermore, the data represent the only information ever collected across the state on game species and hunting. Thus, the data presented here should be viewed as a baseline for actual numbers, a series of patterns that provide a valuable silhouette of the past.

### Management implications

Long term game harvest estimates are essential to all wildlife management agencies (e.g., [20]). Until now, harvest counts in Hawai‘i have focused on the number of animals harvested in recent years, with little to no emphasis on long-term trends. Our analysis provides the first ever information on the rate of removal per island as well as per species, a perspective that is of critical value in formulating management guidelines and policy in Hawai‘i. Such data are fundamental before appropriate research projects can be designed that actually address some of the issues in Hawai‘i, such as the effects of game species density on ecological processes over various types of terrain and where could game management areas be located that minimize ecological degradation. The fact that much of the data (even recently collected data) are incomplete or missing altogether, points to a need for improvements in the type of data collected and data collection methods across the state. Results indicate that game management may be more effective when approached at the local (island) level in Hawai‘i since historical trends for individual species have varied so widely across islands. Taken together, our analyses indicates the need to consider changing the ways in which harvest data are collected and game species are enumerated. Without improvement in data collection and estimation of game species, management, conservation, restoration, and hunting will continue to be challenging across Hawai‘i.

## Supporting information

S1 Table Raw data from P-R reports. (XLSX)
